# Cyclo(phenylalanine‐proline) induces DNA damage in mammalian cells *via* reactive oxygen species

**DOI:** 10.1111/jcmm.12678

**Published:** 2015-09-28

**Authors:** Kwanghyun Lee, Jae Eun Jeong, In Hwang Kim, Kun‐Soo Kim, Bong‐Gun Ju

**Affiliations:** ^1^ Department of Life ScienceSogang UniversitySeoulKorea

**Keywords:** cyclo(phenylalanine‐proline), DNA damage, reactive oxygen species, cyclo dipeptide, comet assay

## Abstract

Cyclo(phenylalanine‐proline) is produced by various organisms such as animals, plants, bacteria and fungi. It has diverse biological functions including anti‐fungal activity, anti‐bacterial activity and molecular signalling. However, a few studies have demonstrated the effect of cyclo(phenylalanine‐proline) on the mammalian cellular processes, such as cell growth and apoptosis. In this study, we investigated whether cyclo(phenylalanine‐proline) affects cellular responses associated with DNA damage in mammalian cells. We found that treatment of 1 mM cyclo(phenylalanine‐proline) induces phosphorylation of H2AX (S139) through ATM‐CHK2 activation as well as DNA double strand breaks. Gene expression analysis revealed that a subset of genes related to regulation of reactive oxygen species (ROS) scavenging and production is suppressed by the cyclo(phenylalanine‐proline) treatment. We also found that cyclo(phenylalanine‐proline) treatment induces perturbation of the mitochondrial membrane, resulting in increased ROS, especially superoxide, production. Collectively, our study suggests that cyclo(phenylalanine‐proline) treatment induces DNA damage *via* elevation of ROS in mammalian cells. Our findings may help explain the mechanism underlying the bacterial infection‐induced activation of DNA damage response in host mammalian cells.

## Introduction

Naturally abundant cyclic dipeptides (2,5‐diketopiperazines, CDPs) are produced by bacteria, fungi, plants and animals 1, 2. CDPs have been shown to be generated by large multimodular nonribosomal peptide synthetases. However, nonribosomal peptide synthetases‐independent cyclic peptides biosynthesis by tRNA‐dependent cyclodipeptide synthases is also found in some bacteria 3, 4. CPDs have diverse biological functions such as anti‐fungal, anti‐bacterial, anti‐viral, anti‐tumour, cell signalling, virulent factor and immunosuppressive activities 5, 6, 7, 8, 9, 10, 11.

Cyclo(phenylalanine‐proline) (cFP) is produced by various bacteria such as *Lactobacillus reuteri*,* Streptomyces* sp. AMLK‐335, *Vibrio vulnificus*,* V. cholera*,* Pseudomonas aeruginosa* and *P. putida* 6, 7, 12, 13, 14, 15. A number of studies have been conducted to investigate the biological function of cFP. For example, cFP demonstrated an anti‐bacterial function against diverse bacteria such as *Escherichia coli* and *P. aeruginosa* 16. In addition, cFP from the *L. plantarum* strain was shown to be anti‐fungal 17. In some bacteria, cFP functions as a quorum‐signal molecule. cFP from *V. vulnificus* was shown to induce the *ompU* gene, which is important for the pathogenicity of *V. vulnificus* 6. In *V. cholera*, cFP attenuates the production of the cholera toxins by activating the expression of the regulatory gene *leuO* 7. The *L. reuteri* RC‐14 also produces cFP, which inhibits the quorum‐sensing system in staphylococci, leading to repression of the expression of staphylococcal exotoxin toxic shock syndrome toxin‐1 in the human vagina 15. CDPs including cFP from *P. aeruginosa* promote growth of *Arabidopsis thaliana* seedlings through activation of auxin‐regulated gene expression 18. These results indicate that cFP may be an evolutionally conserved signalling molecule among bacteria or between prokaryotes and eukaryotes.

A few studies have reported the biological effects of cFP on mammalian cell differentiation and metabolism. When HT‐29 colon adenocarcinoma cells are treated with cFP, cells are differentiated, most likely because of alternation of gene expression *via* increased cAMP response element‐binding protein (CREB) phosphorylation and histone acetylation 5. High concentration of cFP induces cell growth arrest and apoptosis through caspase‐3 activation and Poly ADP ribose polymerase (PARP) cleavage in HT‐29 colon cancer cells 19, 20. Interestingly, treatment of 10 μM cFP isolated from *Streptomyces* sp. AMLK‐335 specifically inhibits DNA topoisomerase I activity, with reduced DNA relaxation in *in vitro* assay, indicating that cFP may be used as an anti‐tumour agent 14. In the present study, we investigated the function of cFP in DNA damage response in mammalian cells. Our results indicate that cFP induces DNA damage such as DNA double strand break (DSB) through elevation of ROS by suppression of a subset of genes related to ROS metabolism as well as perturbation of the mitochondrial membrane in mammalian cells.

## Materials and methods

### Cell lines and chemicals

INT‐407 cell, U2OS cells and Huh7 cells were maintained in DMEM supplemented with 10% foetal bovine serum and antibiotics. Linear phenylalanine‐proline peptides (P6258; Sigma‐Aldrich, St. Louis, MO, USA) and cyclo(phenylalanine‐proline) peptide (G4720; Bachem, Bubendorf, Switzerland) were dissolved in DMEM containing 10% FBS and antibiotics. KU‐55933 (ab120637; Abcam, Cambridge, MA, USA) was dissolved in DMSO. Doxorubicin (D1317; Sigma‐Aldrich) and *N*‐acetylcysteine (A9165; Sigma‐Aldrich) were dissolved in H_2_O. MnTBAP chloride (sc221954; Santa Cruz Biotechnology, Santa Cruz, CA, USA) was dissolved in 0.1 M NaOH.

### Immunocytochemistry

Cells were fixed for 15 min. with 4% paraformaldehyde in PBS and permeabilized with PBST solution (0.5% Triton X‐100 in PBS) for 30 min. After blocking with 5% bovine serum albumin in PBST solution for 1 hr, cells were incubated with the anti‐phospho H2AX (S139) (05‐636; Millipore, Temecula, CA, USA), anti‐53BP1 (sc22760; Santa Cruz Biotechnology), anti‐phospho ATM (S1981) (ab1888; Abcam) antibodies for overnight at 4°C respectively. Antigen was detected with the secondary antibodies conjugated to FITC (F0382; Sigma‐Aldrich) and TRITC (T5393; Sigma‐Aldrich). Cells were coverslipped using VECTASHIELD mounting media plus DAPI (H‐1200; Vector Laboratories, Burlingame, CA, USA). Images were acquired with a confocal microscope (Leica TCS SPE; Leica Microsystems GmbH, Wetzlar, Germany). Percentages of γH2AX foci positive cells were counted in five random high power fields.

### Cell growth analysis

Cell confluence and morphology were monitored automatically at every 4 hrs using IncuCyte imaging system (Essen Bioscience, Ann Arbor, MI, USA).

### Cell death analysis

Cell death was analysed using Annexin V‐FITC Apoptosis Detection Kit I (556547; BD Pharmingen, San Diego, CA, USA) and 7‐AAD (559925; BD pharmingen) according to manufacturer's instruction. Briefly, cells were resuspended in binding buffer (140 mM NaCl, 2.5 mM CaCl_2_, 0.01 M HEPES (4‐(2‐hydroxyethyl)‐1‐piperazinethanesulfonic acid), pH 7.4). Then cells were incubated with Annexin V‐FITC and 7‐AAD for 15 min. at RT in the dark. The stained cells were analysed by flow cytometer (FACSCalibur; BD Bioscience, San Jose, CA, USA) in the FL1 and FL3 channels.

### Western blot analysis

Cells were sonicated in IP150 buffer [10% glycerol, 0.5 mM ethylenediaminetetraacetic acid (EDTA), 25 mM Tris‐HCl, 0.1% NP40 150 mM NaCl and 1 mM Dithiothreitol (DTT), pH 8.0] in the presence of complete protease inhibitors (P5101; GenDEPOT, Barker, TX, USA), 1 mM phenylmethylsulphonylfluoride (20203, USB) and 1 mM sodium orthovanadate (S6508; Sigma‐Aldrich). After clearing by centrifugation, Western blotting was carried out using anti‐phospho H2AX (S139) (05‐636; Millipore), anti‐phospho ATM (S1981) (ab1888; Abcam), anti‐phospho ATR (S428) (sc109912; Santa Cruz Biotechnology), anti‐phospho CHK2 (T68) (04‐1471; Millipore) and anti‐β‐actin (YF‐MA‐10008; ABfrontier, Seoul, Korea) antibodies by standard procedures. Detection of released cytochrome c in the cytoplasm was performed according to previous procedure 21. Briefly, cells were suspended with cold IBcells‐1 buffer [225 mM mannitol, 75 mM sucrose, 0.1 mM EGTA, 30 mM TrisHCl (pH 7.4)] and homogenized by a Teflon pestle with 25 strokes. Cell extract was centrifuged at 600 × g for 5 min. at 4°C. The resulting supernatant was centrifuged at 7000 × g for 10 min. at 4°C. Western blotting was carried out using an anti‐cytochrome c (ab13575; Abcam) antibody. Western blots were analysed quantitatively using the ImageJ software (NIH, Bethesda, MD, USA). The band intensity was normalized against β‐actin and band intensity of control considered as 1.

### Neutral comet assay

Cells were combined with 1% low melting agarose at a ratio of 1:10 (v/v) and immediately pipetted on to slide glass. The slide glasses were placed at 4°C for 30 min. and immersed in cold lysis solution (2% sarkosyl, 0.5 M EDTA, 0.5 mg/ml proteinase K, pH 8.0) at 4°C for overnight. After slide glass was washed with 1× Tris Borate EDTA (TBE) buffer at 4°C for 30 min., electrophoresis was performed for 40 min. at 0.6 V/cm. The slide glass was washed with DIW for 5 min. and 70% Ethanol for 5 min. The slide glass was dried and stained with propidium iodide (P4170, Sigma‐Aldrich). DNA damage was analysed by examining at least 50 comets using the Comet Score program (TriTek Corporation, Sumerduck, VA, USA).

### RNA interference (siRNA)

INT‐407 cells were transfected with siRNA against human *ATM* (SV 1002; Bioneer, Daejeon, Korea) or control siRNA (sc37007; Santa Cruz Biotechnology) using an XtreamGENE siRNA transfection reagent (Roche, Mannheim, Germany). The efficiency of knock down of specific gene was confirmed with real‐time PCR.

### RNA‐Seq

Total RNA was extracted using RNeasy mini kit (Qiagen, Valencia, CA, USA). The quality of the total RNA was evaluated using RNA electropherogram (Experion; Bio‐Rad, Hercules, CA, USA) and assessing the RNA quality indicator. The resulting mRNA samples were processed for the sequencing libraries using the Illumina TruSeq Stranded mRNA sample preparation kit (Illumina, San Diego, CA, USA) following the manufacturer's protocols. One lane per 6 samples was used for sequencing by the Illumina HiSeq 2500 to generate directional, paired‐end 100‐base‐pair reads. Quality‐filtered reads were mapped to the human reference genome sequences, hg19 (UCSC Genome Bioinformatics, https://genome.ucsc.edu) using tophat2 (http://ccb.jhu.edu/software/tophat) 22. The relative transcript abundance was estimated by counting the fragments per kilobase of exon model per million mapped sequence reads (FPKM) and differential expressed genes were evaluated using cufflinks package (http://cufflinks.cbcb.umd.edu) 23. Gene Ontology categories with differential expressed genes were analysed by DAVID (http://david.abcc.ncifcrf.gov).

### Real‐time RT‐PCR

First strand cDNA synthesis from total RNA template was performed with PrimeScript II 1st strand cDNA Synthesis Kit (Takara Bio, Otsu, Shiga, Japan). The resulting cDNAs were subjected to real‐time PCR with a Stratagene Mx3000P (Agilent Technologies, Santa Clara, CA, USA) using TOP SYBR Green (Enzynomics, Daejeon, Korea). PCR conditions used to amplify all genes were 10 min. at 95°C and 40 cycles of 95°C for 15 sec., 60°C for 40 sec. Expression data were calculated from the cycle threshold (Ct) value using the ΔCt method for quantification. *GAPDH* mRNA levels were as used for normalization. Oligonucleotides were described in Table S1.

### ROS measurement

After cells were harvested with trypsin‐EDTA and washed with PBS, cells were stained with 5 μM dichlorofluorescein diacetate (DCFDA; D6883; Sigma‐Aldrich) for 90 min. at 37°C or 5 M MitoSox Red (M36008; Molecular Probes, Eugene, OR, USA) for 30 min. at 37°C in the dark. Flow cytometry was performed with flow cytometer (FACSCalibur; BD Bioscience, San Jose, CA, USA) in the FL1 channel for DCFDA or FL3 channel for MitoSox Red. For cell imaging, cells were stained with 5 μM MitoSox Red for 30 min. at 37°C in the dark. After cells were fixed for 15 min. with 4% paraformaldehyde in PBS, cells were permeabilized with PBST solution (0.5% Triton X‐100 in PBS) for 30 min. in the dark. Cells were coverslipped using VECTASHIELD mounting media plus DAPI. Images were acquired with a confocal microscope (Leica TCS SPE; Leica).

### Mitochondria membrane potential measurement

Cells were stained with 5 μM Rhodamine 123 (R8004; Sigma‐Aldrich) for 30 min. at 37°C in the dark. Flow cytometry was performed with Flow cytometer (FACSCalibur; BD Bioscience, San Jose, CA, USA) in the FL1 channel.

### Statistical analyses

All quantitative data are presented as mean ± S.D. for three independent experiments. anova was used for multiple comparisons. Significance values were *P ≤ 0.05, **P ≤ 0.01 and ***P ≤ 0.005.

## Results

### Induction of DNA damage by cFP treatment in mammalian cells

**Figure 1:**
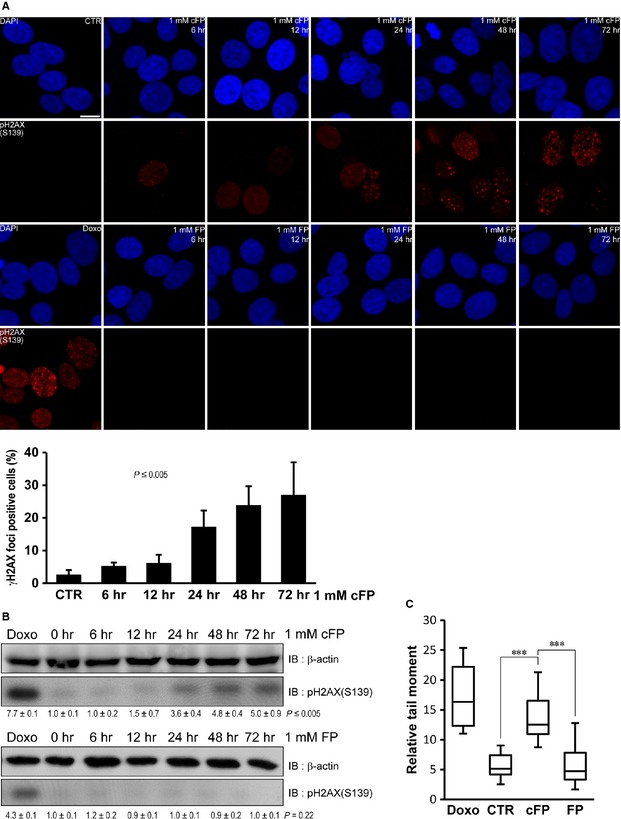
Induction of DNA damage in cFP‐treated INT‐407 cells. (**A**) Representative images demonstrate the increased γH2AX foci formation in cFP‐treated INT‐407 cells. In contrast, 1 mM linear FP dipeptides (FP) treatment or no treatment (CTR) does not induce γH2AX foci formation. After INT‐407 cells were incubated with 1 mM cFP for indicated times, cells were immunostained with an anti‐phospho H2AX (S139) antibody. Nuclei were identified using DAPI staining. As a positive control, INT‐407 cells were treated with 1 μM doxorubicin (Doxo); scale bar, 10 μm. (**B**) Phosphorylation of H2AX (S139) is increased in cFP‐treated INT‐407 cells. However, treatment of 1 mM linear FP dipeptides (FP) does not induce H2AX (S139) phosphorylation. As positive control, INT‐407 cells were treated with 1 μM doxorubicin (Doxo). The lysates were immunoblotted with anti‐phospho H2AX (S139) antibody. The anti‐β actin antibody was used as a loading control. Western blots were analysed quantitatively. Relative band intensities are expressed as the mean ± S.D. (**C**) Increased DNA damage in cFP‐treated INT‐407 cells. After cells were incubated with 1 mM cFP or linear FP dipeptides (FP) for 48 hrs, cells were embedded, lysed and electrophoresed in agarose. DNA damage was analysed using the Comet Score program. As controls, INT‐407 cells were untreated (CTR) or treated with 1 μM doxorubicin (Doxo). ***P ≤ 0.005.

cFP is produced from bacteria, fungi, plants and animals. It may function as an anti‐cancer agent by induction of apoptosis or inhibition of DNA topoisomerase I 5, 14, 19, 20. To investigate the possibility that cFP treatment can induce DNA damage response in mammalian cells. We chose HeLa derivative INT‐407 cell line as *in vitro* model because it has been used in the studies on DNA damage and pathogenesis of *Vibrio* sp., which produces cFP 6, 7, 24, 25, 26, 27, 28, 29, 30, 31. We first measured the level of DNA damage with γH2AX foci formation using an anti‐phospho H2AX (S139) antibody. We observed cFP‐induced accumulation of γH2AX foci in concentration and incubation time‐dependent manner (Fig. 1A and Fig. S1). In particular, γH2AX foci formation was detected readily at 48 hrs after 1 mM cFP treatment. In contrast, untreated control (CTR) or linear Phe‐Pro dipeptides (FP) did not induce γH2AX foci formation in any tested concentrations or incubation times (Fig. 1A). As a positive control, INT‐407 cells were treated with 1 μM doxorubicin (Doxo), a DNA damage‐inducing agent. To exclude the cell line specific response to cFP treatment, several mammalian cells including U2OS osteosarcoma and Huh7 hepatoma cells were tested. Although there was some variation in concentration and incubation time for cFP treatment, we consistently observed cFP‐induced γH2AX foci formation (Fig. S2). We further confirmed cFP‐induced DNA damage by Western blot analysis. While phosphorylation of H2AX (S139) was barely detected in linear FP dipeptides‐treated INT‐407 cells, increased phosphorylation of H2AX (S139) was observed at 48 hrs after 1 mM cFP treatment (Fig. 1B). To detect the physical DSB in cFP‐treated INT‐407 cells, a neutral comet assay was performed. As expected, we found a significant increase in DSB in 1 mM cFP‐treated cells (Fig. 1C). However, we did not observe increased DSB in linear FP dipeptides‐treated cells. Collectively, these results suggest that cFP treatment induces DNA damage such as DSB in mammalian cells.

### Activation of DNA damage response in cFP‐treated INT‐407 cells

**Figure 2:**
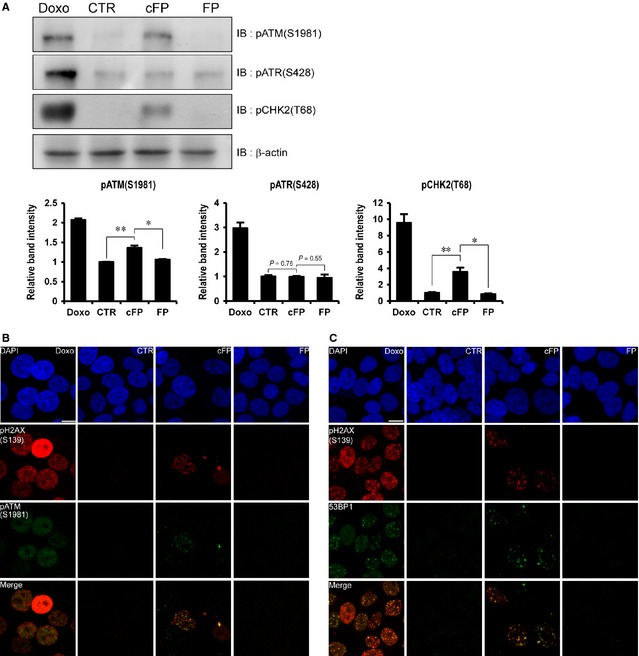
Activation of DNA damage response in cFP‐treated INT‐407 cells. (**A**) CHK2 (T68) and ATM (S1981) are phosphorylated in cFP‐treated INT‐407 cells. After cells were incubated with 1 mM cFP or linear FP dipeptides (FP) for 48 hrs, the lysates were immunoblotted with anti‐phospho ATR (S428), anti‐phospho ATM (S1981) and anti‐phospho CHK2 (T68) antibodies respectively. As controls, INT‐407 cells were untreated (CTR) or treated with 1 μM doxorubicin (Doxo). Western blots were analysed quantitatively. (**B** and **C**) Representative images demonstrate the co‐localization of phosphorylated H2AX (S139), phosphorylated ATM (S1981) and 53BP1 in cFP‐treated INT‐407 cells. The cells treated with 1 mM cFP or linear FP dipeptides (FP) for 48 hrs were co‐immunostained with anti‐phospho H2AX (S139), anti‐phospho ATM (S1981) and anti‐53BP1 antibodies in combination. As controls, INT‐407 cells were untreated (CTR) or treated with 1 μM doxorubicin (Doxo); scale bar, 10 μm. *P ≤ 0.05 and **P ≤ 0.01.

DNA damage triggers a cellular signalling pathway that regulates the cell cycle and DNA damage repair through either ATR‐CHK1 or ATM‐CHK2 activation. Therefore, we next checked ATR and ATM activation in 1 mM cFP‐treated INT‐407 cells using anti‐phospho ATR (S428) and anti‐phospho ATM (S1981) antibodies respectively. Western blot analysis revealed that phosphorylation level of ATR (S428) did not change by cFP treatment as compared to untreated control (CTR) or linear Phe‐Pro dipeptides (FP) treatment (Fig. 2A). However, we observed increased phosphorylation of ATM (S1981) and CHK2 (T68), indicating that cFP‐induced DNA damage response is occurred through the activation of ATM‐CHK2 signalling pathway. In addition, we did not observe phosphorylation of ATM and CHK2 in FP dipeptides‐treated cells (Fig. 2A). We further confirmed the activation of ATM by co‐localization of phosphorylated ATM (S1981) with γH2AX foci in 1 mM cFP‐treated INT‐407 cells (Fig. 2B). We also observed co‐localization of the 53BP1 checkpoint protein with γH2AX foci in 1 mM cFP‐treated cells (Fig. 2C).

### Requirement of ATM in cFP‐induced DNA damage response

**Figure 3:**
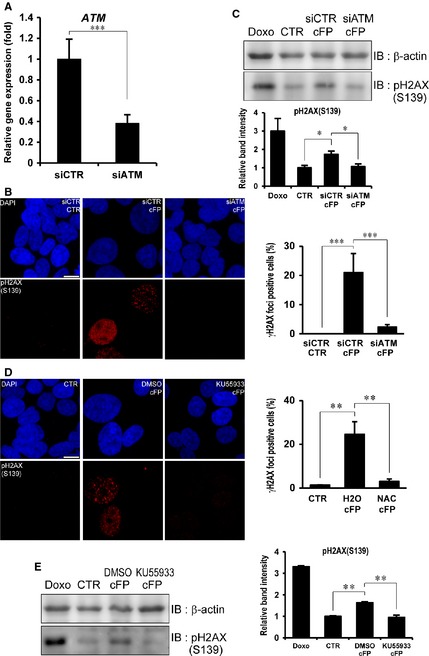
ATM‐mediated DNA damage in cFP‐treated INT‐407 cells. (**A**) The transcript of *ATM* was efficiently depleted by *ATM* siRNA. The expression of ATM was determined by real‐time PCR in control (siCTR) or *ATM* siRNA (siATM). (**B**) Representative images demonstrate that depletion of *ATM* decreases γH2AX foci formation in cFP‐treated INT‐407 cells. After control or *ATM* siRNA transfection, cells were incubated with 1 mM cFP for 48 hrs and immunostained with an anti‐phospho H2AX (S139) antibody. Untreated cells (CTR) were used as a negative control; scale bar, 10 μm. (**C**) Depletion of *ATM* decreases phosphorylation of H2AX (S139) in cFP‐treated INT‐407 cells. After siRNA transfection, cells were incubated with 1 mM cFP for 48 hrs and lysates were immunoblotted with anti‐ phospho H2AX (S139) antibody. As controls, INT‐407 cells were untreated (CTR) or treated with 1 μM doxorubicin (Doxo). Western blots were analysed quantitatively. (**D**) Representative images demonstrate that enzyme activity of ATM is required for cFP‐induced γH2AX foci formation. After cells were incubated with 1 mM cFP in conjunction with 10 μM ATM inhibitor (KU‐55933) or DMSO for 48 hrs, cells were immunostained with anti‐ phospho H2AX (S139) antibody. Untreated cells (CTR) were used as a negative control; scale bar, 10 μm. (**E**) After cells were incubated with 1 mM cFP in conjunction with 10 μM ATM inhibitor (KU‐55933) or DMSO for 48 hrs, the lysates were immunoblotted with anti‐ phospho H2AX (S139) antibody. As controls, INT‐407 cells were untreated (CTR) or treated with 1 μM doxorubicin (Doxo). Western blots were analysed quantitatively. *P ≤ 0.05, **P ≤ 0.01 and ***P ≤ 0.005.

Next, we tested whether cFP‐induced DNA damage is dependent on ATM kinase, which functions as a DNA damage sensor and phosphorylates H2AX (S139). We first depleted expression of *ATM* by siRNA in INT‐407 cells, and cells were treated with 1 mM cFP (Fig. 3A). Using *ATM*‐depleted INT‐407 cells, the level of DNA damage was determined with γH2AX foci formation after 1 mM cFP treatment for 48 hrs. We found decreased γH2AX foci formation in 1 mM cFP‐treated cells as compared to control siRNA‐transfected cells (Fig. 3B). We further confirmed a decreased level of H2AX (S139) phosphorylation by Western blot analysis (Fig. 3C). Given that kinase activity of ATM is critical for the DNA damage sensor and H2AX (S139) phosphorylation, we treated INT‐407 cells with 1 mM cFP in conjunction with 10 μM ATM inhibitor (KU‐55933) or DMSO solvent. As with *ATM* depletion by siRNA, we found decreased γH2AX foci formation by immunocytochemistry (Fig. 3D) and decreased H2AX phosphorylation (S139) by Western blot analysis (Fig. 3E). Collectively, these results suggest that ATM is required for cFP‐induced DNA damage response such as H2AX (S139) phosphorylation.

### Treatment of cFP alters the gene expression related to ROS production and scavenging

**Figure 4:**
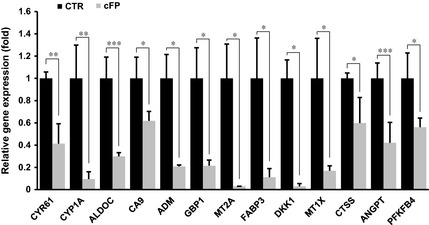
Expression of genes related to ROS scavenging and production in cFP‐treated INT‐407 cells. After INT‐407 cells were incubated with 1 mM cFP for 48 hrs, gene expression was determined by real‐time PCR. Relative expression was determined by comparing gene expression between untreated (CTR) and 1 mM cFP‐treated INT‐407 cells. Transcript of *GAPDH* was used as control. *P ≤ 0.05, **P ≤ 0.01 and ***P ≤ 0.005.

To gain insight into the molecular mechanism underlying cFP‐induced DNA damage response in INT‐407 cells, RNA‐seq was performed with total RNA from 1 mM cFP‐treated or ‐untreated INT‐407 cells. A relatively small number of genes (38) were regulated in cFP‐treated INT‐407 cells (8 up‐regulated and 30 down‐regulated) as compared to untreated cells. Table 1 shows the top 8 up‐regulated and 10 down‐regulated genes. When 38 regulated genes were analysed using GO (gene ontology), we found that some of the genes participated in GO including response to hypoxia and oxygen level (Table 1). Interestingly, some of the down‐regulated genes such as *MT1X*,* MT2A*,* ADM*,* ANGPTL4*,* CTSS*,* CYP1A1*,* PFKFB4*,* FABP3* and *DKK1* are involved in regulation of ROS scavenging and production 32, 33, 34, 35, 36, 37, 38, 39, 40, 41. We confirmed the result from RNA‐seq by real‐time PCR (Fig. 4). These results imply that cFP may induce DNA damage by suppression of genes related to regulation of ROS scavenging and production in mammalian cells.

**Table 1:** Up‐ or down‐regulated genes in cFP‐treated INT‐407 cells

Gene	Reads	log_2_ (fold change)	*P*‐value	GO pathway
*RANBP3L*	10.9941	2.54324	5.00E‐05	Transport
*COL15A1*	20.4543	2.42053	5.00E‐05	Angiogenesis Blood vessel morphogenesis Blood vessel development
*MUC16*	7.37539	2.11018	5.00E‐05	Cell adhesion
*NR4A2*	7.95682	2.07375	0.00065	Response to hypoxia Response to oxygen levels Response to inorganic substance
*RPPH1*	110.345	2.03445	0.00085	Response to stimulus
*ALDH1L2*	2.03469	2.01453	0.0008	Response to drug
*PCLO*	2.02115	1.57126	0.0008	Regulation of hormone levels
*NR4A1*	46.9685	1.36902	0.00085	Response to hypoxia Response to oxygen levels
*MT2A*	146.666	−1.61239	0.0002	Response to immune system
*ACSS2*	23.8998	−1.79115	5.00E‐05	Lipid biosynthetic process
*CYR61*	16.8891	−1.8206	5.00E‐05	Angiogenesis Blood vessel morphogenesis Blood vessel development
*PFKFB4*	1.99561	−2.04262	0.00045	Fructose metabolic process
*ALDOC*	12.4805	−2.35588	5.00E‐05	Response to hypoxia Response to oxygen levels
*CYP1A1*	2.74474	−2.41925	5.00E‐05	Response to hypoxia Response to oxygen levels Isoprenoid metabolic process Isoprenoid biosynthetic process Response to inorganic substance Terpenoid metabolic process Lipid biosynthetic process
*CA9*	2.95148	−2.65043	5.00E‐05	Response to hypoxia Response to oxygen levels
*ADM*	5.2022	−2.83732	5.00E‐05	Response to hypoxia Response to oxygen levels Lipid biosynthetic process
*MT1X*	13.2878	−3.09664	5.00E‐05	Response to inorganic substance
*GBP1*	1.72728	−3.21058	5.00E‐05	Response to immune system

### ROS‐dependent DNA damage in cFP‐treated INT‐407 cells

**Figure 5:**
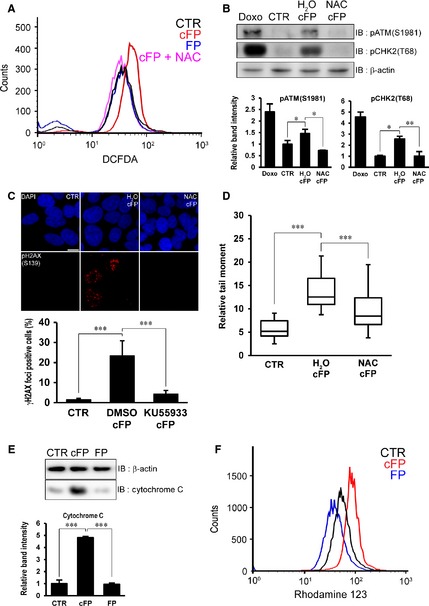
ROS‐dependent DNA damage in cFP‐treated INT‐407 cells (**A**) The level of ROS is elevated by 1 mM cFP treatment in INT‐407 cells. However, 1 mM linear FP dipeptides (FP) treatment does not elevate level of ROS. After cells were incubated with 1 mM cFP in conjunction with 0.5 mM *N*‐acetylcysteine (NAC) or H_2_O for 48 hrs, ROS level was determined by flow cytometry using DCFDA. Untreated cells (CTR) were used as a negative control. (**B**) ROS scavenger treatment decreases cFP‐induced phosphorylation of CHK2 (T68) and ATM (S1981). The lysates were immunoblotted with anti‐phospho CHK2 (T68) and anti‐phospho ATM (S1981) antibodies respectively. As controls, INT‐407 cells were untreated (CTR) or treated with 1 μM doxorubicin (Doxo). Western blots were analysed quantitatively. (**C**) Representative images demonstrate that ROS scavenger treatment decreases cFP‐induced γH2AX foci formation. After cells were incubated with 1 mM cFP in conjunction with 0.5 mM NAC or H_2_O for 48 hrs, cells were immunostained with anti‐ phospho H2AX (S139) antibody. Untreated cells (CTR) were used as a negative control; scale bar, 10 μm. (**D**) ROS scavenger treatment attenuates cFP‐induced DNA damage. After cells were incubated with 1 mM cFP in conjunction with 0.5 mM NAC or H_2_O for 48 hrs, a neutral comet assay was performed. Untreated cells (CTR) were used as a negative control. (**E**) Increased cytochrome C release in cFP‐treated INT‐407 cells. The cytoplasmic fraction from 1 mM cFP or linear FP dipeptides‐treated INT‐407 cells was immunoblotted with an anti‐cytochrome C antibody as described in Materials and Methods section. Untreated cells (CTR) were used as a negative control. Western blots were analysed quantitatively. (**F**) Disturbance of mitochondrial membrane is induced in cFP‐treated INT‐407 cells. After INT‐407 cells were treated with 1 mM cFP or linear FP dipeptides for 48 hrs, cells were stained with 5 μM Rhodamine 123 and flow cytometry was performed. Untreated cells (CTR) were used as a negative control. *P ≤ 0.05, **P ≤ 0.01 and ***P ≤ 0.005.

Because cFP treatment suppresses a set of genes related to regulation of ROS scavenging and production in INT‐407 cells, we determined ROS level in cFP‐treated cells by flow cytometry. After INT‐407 cells were treated with 1 mM cFP for 6, 12, 24, 48, 72 hrs, cells were further incubated with DCFDA for 90 min. and flow cytometry was performed. While we did not detect significant ROS production in untreated or FP dipeptides‐treated cells, 1 mM cFP‐induced ROS production in an incubation time‐dependent manner (Fig. 5A and Fig. S3A, compare the black, blue and red lines). We also found that ROS is produced before DNA damage is occurred, which is demonstrated by phosphorylation of CHK2 (T68), ATM (S1981) and H2AX (S139) (Fig. 5B and Fig. S3B). When cells were co‐treated with 1 mM cFP and 0.5 mM *N*‐acetylcysteine (NAC) ROS scavenger, increased ROS production by cFP treatment was abolished (Fig. 5A, compare the red and pink lines). Since cFP‐induced ROS production and DNA damage response, we investigated whether NAC treatment attenuates DNA damage response by Western blot analysis. We found decreased DNA damage response, which is demonstrated by decreased phosphorylation of CHK2 (T68) and ATM (S1981; Fig. 5B). Immunocytochemical results further confirmed that NAC treatment results in decreased γH2AX foci formation (Fig. 5C). Finally, a neutral comet analysis confirmed that cFP‐induced physical DSB formation is attenuated significantly by removal of ROS (Fig. 5D).

Since the mitochondrial respiratory chain is the main source of ROS production and superoxide is predominant ROS in mitochondria 42, 43, 44, 45, we used MnTBAP, superoxide dismutase (SOD) mimetic, to reduce level of superoxide in cFP‐treated cells 46, 47. To detect superoxide, MiotSox Red reagent also was used for flow cytometric anlysis and cell imaging. MnTBAP treatment efficiently reduced cFP‐induced superoxide from mitochondria (Fig. S4A). In addition, MnTBAP attenuated cFP‐induced DNA dmage response, which is assessed by immunocytochemistry, Western blotting, and neutral comet assay (Fig. S4B–D). By measuring cytochrome C release and membrane potential, we further tested whether cFP impairs the mitochondria membrane. As expected, cFP treatment induced increased cytochrome C release (Fig. 5E) and disturbance of mitochondria potential (Fig. 5F). These results indicate that cFP may also induce DNA damage *via* ROS, especially superoxide, production in the mitochondria.

**Figure 6:**
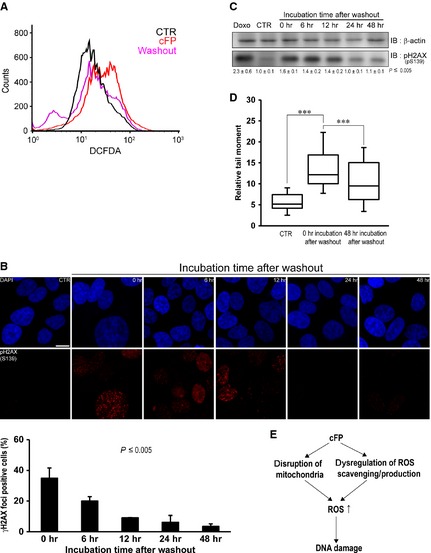
Removal of cFP restores DNA damage repair in INT‐407 cells. After INT‐407 cells were incubated with 1 mM cFP for 48 hrs, cell culture media were changed with new media without cFP and cultured for the indicated times. (**A**) Washout of cFP attenuates ROS production in cFP‐treated INT‐407 cells. ROS level was determined by flow cytometry. Untreated cells (CTR) were used as a negative control. (**B**) Representative images demonstrate that washout of cFP decreases γH2AX foci formation in cFP‐treated INT‐407 cells. Cells were immunostained with anti‐phospho H2AX (S139) antibody. Untreated cells (CTR) were used as a negative control; scale bar, 10 μm. (**C**) Washout of cFP decreases H2AX (S139) phosphorylation in cFP‐treated INT‐407 cells. The lysates were immunoblotted with anti‐phospho H2AX (S139) antibody. As controls, INT‐407 cells were untreated (CTR) or treated with 1 μM doxorubicin (Doxo). Western blots were analysed quantitatively. Relative band intensities are expressed as the mean ± S.D. (**D**) Washout of cFP attenuates DNA damage in cFP‐treated INT‐407 cells. A neutral comet assay after wash was performed. Untreated cells (CTR) were used as a negative control. (**E**) A proposed model. cFP treatment induces down‐regulation of genes related to ROS production/scavenging and increased ROS (superoxide) production in the mitochondria. This event may maintain the elevated level of ROS (superoxide), leading to DNA damage such as DSB. *P ≤ 0.005.

We next tested whether DNA damage repair is restored after washout of cFP in a cell culture. After INT‐407 cells were treated with 1 mM cFP for 48 hrs, cell culture media was changed with new media without cFP and cultured for the indicated times. We found that washout of cFP decreases ROS production in INT‐407 cells (Fig. 6A, compare the red and pink lines). In addition, immunocytochemistry clearly showed a decreased number of γH2AX foci formation at 24 hrs after washout of cFP (Fig. 6B). Consistently, Western blot analysis indicated decreased phosphorylation of H2AX (S139) after washout of cFP (Fig. 6C). We further examined whether DNA damage repair would restore after washout of cFP by a neutral comet assay. Parallel with the above results, DNA damage was significantly decreased (Fig. 6D). Interestingly, INT‐407 cells treated with 1 mM cFP for 120 hrs retained the DNA damage repair capacity (Fig. S5). However, more than 8 mM cFP treatment for 48 hrs failed to restore DNA damage repair, showing decreased survived cells (Fig. S6A). Consistently, flow cytometric analysis demonstrated that 8 mM cFP treatment increases cell necrosis (Fig. S6B).

Collectively, we demonstrated that 1 mM cFP induces DNA damage such as DSB through elevation of ROS by suppression of a subset of genes related to ROS metabolism as well as perturbation of the mitochondrial membrane in mammalian cells.

## Discussion

Naturally abundant cFP has various biological functions such as anti‐bacterial activity, anti‐fungal activity, quorum sensing, bacterial virulence, biofilm formation and plant auxin production 5, 6, 7, 8, 9, 10, 11. However, a few studies have investigated the biological effects of cFP on mammalian cells. For example, cFP treatment induces cell growth arrest and apoptosis through caspase‐3 activation and PARP cleavage in HT‐29 colon cancer cells 19, 20. In addition, cFP induces cell differentiation in HT‐29 cells 48. Since cFP inhibits DNA Topoisomerase I activity based on an *in vitro* DNA relaxation assay 14, it would be interesting whether cFP induces DNA damage response in mammalian cells. In this study, we found that 1 mM cFP induces activation of DNA damage response such as CHK2/ATM activation. Furthermore, it induced DSB as determined by H2AX (S139) phosphorylation and a neutral comet assay. Although concentration of cFP tested in this study was relatively high, millimolar cFP seems to be the physiological concentration. In fact, some bacteria such as *V. vulnificus* produces approximately 1 mM cFP in culture 6. In addition, millimolar concentration of cFP regulates diverse biological events such as quorum sensing, virulence factor production, auxin signalling, cell growth and differentiation in bacteria, plant and mammalian cells 6, 7, 15, 18, 19, 20, 48. However, more than 8 mM cFP treatment induced cell death. We also found that cFP induces DNA damage in various mammalian cells including INT‐407, Huh7 and U2OS cells with different response kinetics. We believe that this variation may be caused by different cellular origins and contextures. Similarly, it has been reported that doxorubicin treatment induces DNA damage in different cell lines with different incubation time and concentration 49, 50, 51.

Our study further suggests that DSB was not formed immediately but slowly accumulated within 48 hrs after 1 mM cFP treatment, indicating that cFP may alter cell metabolism rather than inducing DSB directly. In addition, RNA‐seq. analysis demonstrated that cFP treatment may induce DNA damage *via* elevated ROS production and/or decreased ROS scavenging. Most of the down‐regulated genes such as *MT1X, MT2A, ADM, CTSS, PFKFB4, FABP3* and *DKK1* are involved in response to hypoxia and oxygen level and DNA damage. Among them, it has been reported that metallothioneins such as MT1X and MT2A protect against ROS toxicity by ROS scavenging activity in plant and animal cells 52, 53, 54, 55, 56. Adrenomedullin (ADM) also has antioxidant activity in angiotensin II‐induced ROS generation in vascular smooth muscle cells 57. This effect may be because of the increased activities of glutathione peroxidase and reductase and maintained total and active reduced thioredoxin levels 34. In addition, when Cathepsin S (CTSS) was inhibited, autophagy and intracellular ROS were induced in HONE1 nasopharyngeal carcinoma cells 36. Depletion of 6‐phosphofructo‐2‐kinase/fructose‐2,6‐biphosphatase 4 (PFKFB4) resulted in a significant decrease in the levels of Dihydronicotinamide‐adenine dinucleotide phosphate (NADPH) that maintain cellular stores of reduced glutathione antioxidant, leading to increased accumulation of ROS in prostate cancer cells 38. Furthermore, depletion of Fatty acid binding protein 3 (FABP3) enhanced ROS production in P19 embryonic carcinoma cells 39. Depletion of Dickkopf WNT signaling pathway inhibitor 1 (DKK1), an antagonist of Wnt/β‐catenin signalling, induced intracellular ROS because of up‐regulation of *ROMO1* (*ROS modulator* 1) expression in A549 cells 40. Although decrease in some genes such as *CYP1A1, ANGPTL4* and *CCN1* resulted in the reduction of ROS production in specific situations 35, 58, 59, we have suggested that 1 mM cFP treatment may increase intracellular ROS production by dysregulation of genes related to ROS scavenging and production.

Consistent with RNA‐seq results, cFP treatment indeed resulted in increased ROS production. In addition, elevated ROS was observed before DNA damage is occurred by cFP treatment (Fig. 5A and Fig. S3). Accumulating evidence indicates that ROS induces oxidative DNA damage by formation of oxidative DNA base adducts including 8‐oxoG, leading to DSB formation 60, 61, 62. Although cFP down‐regulated genes related to regulation of ROS scavenging and production in mammalian cells, we further found that cFP treatment triggers mitochondria perturbation, which results in ROS production. In fact, it has been known that mitochondrial respiratory chain is the main source of ROS production and superoxides are predominant ROS in mitochondria 42, 43, 44, 45. Consistently, we found that MnTBAP, SOD mimetic, treatment abolishes cFP‐induced DNA damage, indicating that superoxide may be the main ROS induced by cFP treatment (Fig. S4). However, we cannot rule out the possible existence of ROS‐independent DSB formation by cFP treatment. For example, cFP inactivates the activity of DNA topoisomerase I that relaxes DNA supercoiling during replication and transcription 14. In the case of the camptothecin DNA topoisomerase I inhibitor, it forms a cleavage complex and prevents the DNA re‐ligation step, leading to DNA single strand breaks (SSBs) 63, 64. This cleavage complex collides with the replication fork during the S phase, resulting in conversion of SSB into DSB 65.

Given that millimolar cFP is produced in many pathogenic bacteria including *V. vulnificus*, and the fact that it plays an important role in regulation of virulence and biofilm formation, which may affect bacteria‐host interaction 6, 7, 66, it is tempting to speculate that cFP itself functions as a virulence factor. Several reports have demonstrated that bacterial infections or pathogens induce DSB in host mammalian cells. For example, infection of pathogenic *Escherichia coli* of phylogenetic group B2 expressing a putative hybrid peptide‐polyketide colibactin induces DSB and activation of DNA damage response, resulting in cell cycle arrest and eventually host cell death 67. Similarly, *Mycoplasma pneumoniae* infection induces changes of protein expression related to oxidative stress and ROS production, leading to DSB in A549 cells 68. In addition, *Chlamydia trachomatis* infection results in ROS production, ERK‐dependent DSB formation and malignant transformation 69. In contrast, *Helicobacter pylori* infection induces ROS‐independent and ATM‐dependent DSB formation, leading to genomic instability and gastric carcinogenesis, although no virulence factor or DSB inducing factor has been identified 70.

Collectively, we demonstrate that cFP induces elevation of ROS, especially superoxide by perturbation of mitochondria membrane as well as dysregulation of a subset of genes related to ROS scavenging and production. In turn, this event results in DNA damage such as DSB in mammalian cells (Fig. 6E). Our findings may explain the bacterial infection‐induced activation of DNA damage response or carcinogenesis in host mammalian cells. However, it is necessary to identify the mechanism underlying cFP uptake, signalling pathway and interacting partners for the clarification of the biological function of cFP. In addition, it is important to develop bacteria that cannot synthesize cFP by destroying responsible genes such as nonribosomal peptide synthetases or tRNA‐dependent cyclodipeptide synthases to test our hypothesis.

## Conflicts of interest

The authors confirm that there are no conflicts of interest.

## Author contribution

Conceived and designed the experiments: KL KK BJ. Performed the experiments: KL JJ. Analysed the data: KL JJ IK KK BJ. Contributed reagents/materials/analysis tools: KK. Wrote the paper: KK BJ.

## Supporting information


**Figure S1** Induction of DNA damage in cFP‐treated INT 407 cells.

**Figure S2** Induction of DNA damage in 5 or 20 mM cFP‐treated U2OS osteosarcoma cells and Huh7 hepatoma cells respectively.

**Figure S3** Incubation time‐dependent ROS production and DNA damage in 1 mM cFP‐treated INT‐407 cells.

**Figure S4** Superoxide‐dependent DNA damage in 1 mM cFP‐treated INT‐407 cells.

**Figure S5** Incubation time‐independent restoration of DNA damage repair after removal of cFP.

**Figure S6** Dose‐dependent restoration of DNA damage repair after removal of cFP.

**Table S1** Oligonucleotides used in this study for real‐time PCR.

## References

[1] Prasad C . Bioactive cyclic dipeptides. Peptides. 1995; 16: 151–64. 7716068 10.1016/0196-9781(94)00017-z

[2] Huang R , Zhou X , Xu T , *et al* Diketopiperazines from marine organisms. Chem Biodivers. 2010; 7: 2809–29. 21161995 10.1002/cbdv.200900211

[3] Belin P , Moutiez M , Lautru S , *et al* The nonribosomal synthesis of diketopiperazines in tRNA‐dependent cyclodipeptide synthase pathways. Nat Prod Rep. 2012; 29: 961–79. 22751625 10.1039/c2np20010d

[4] Giessen TW , Marahiel MA . The tRNA‐dependent biosynthesis of modified cyclic dipeptides. Int J Mol Sci. 2014; 15: 14610–31. 25196600 10.3390/ijms150814610 PMC4159871

[5] Graz CJ , Grant GD , Brauns SC , *et al* Cyclic dipeptides in the induction of maturation for cancer therapy. J Pharm Pharmacol. 2000; 52: 75–82. 10716606 10.1211/0022357001773535

[6] Park DK , Lee KE , Baek CH , *et al* Cyclo(Phe‐Pro) modulates the expression of ompU in Vibrio spp. J Bacteriol. 2006; 188: 2214–21. 16513751 10.1128/JB.188.6.2214-2221.2006 PMC1428137

[7] Bina XR , Bina JE . The cyclic dipeptide cyclo(Phe‐Pro) inhibits cholera toxin and toxin‐coregulated pilus production in O1 El Tor *Vibrio cholerae* . J Bacteriol. 2010; 192: 3829–32. 20453095 10.1128/JB.00191-10 PMC2897329

[8] de Carvalho MP , Abraham WR . Antimicrobial and biofilm inhibiting diketopiperazines. Curr Med Chem. 2012; 19: 3564–77. 22709011 10.2174/092986712801323243

[9] Minelli A , Grottelli S , Mierla A , *et al* Cyclo(His‐Pro) exerts anti‐inflammatory effects by modulating NF‐κB and Nrf2 signalling. Int J Biochem Cell Biol. 2012; 44: 525–35. 22185821 10.1016/j.biocel.2011.12.006

[10] Kwak MK , Liu R , Kwon JO , *et al* Cyclic dipeptides from lactic acid bacteria inhibit proliferation of the influenza A virus. J Microbiol. 2013; 51: 836–43. 24385362 10.1007/s12275-013-3521-y

[11] Kumar SN , Nambisan B , Mohandas C . Purification and identification of two antifungal cyclic dipeptides from Bacillus cereus subsp. thuringiensis associated with a rhabditid entomopathogenic nematode especially against Fusarium oxysporum. J Enzyme Inhib Med Chem. 2014; 29: 190–7. 23402421 10.3109/14756366.2013.765414

[12] Holden MT , Ram Chhabra S , de Nys R , *et al* Quorum‐sensing cross talk: isolation and chemical characterization of cyclic dipeptides from *Pseudomonas aeruginosa* and other gram‐negative bacteria. Mol Microbiol. 1999; 33: 1254–66. 10510239 10.1046/j.1365-2958.1999.01577.x

[13] Degrassi G , Aguilar C , Bosco M , *et al* Plant growth‐promoting Pseudomonas putida WCS358 produces and secretes four cyclic dipeptides: cross‐talk with quorum sensing bacterial sensors. Curr Microbiol. 2002; 45: 250–4. 12192521 10.1007/s00284-002-3704-y

[14] Rhee KH . Inhibition of DNA topoisomerase I by cyclo(L‐Prolyl‐L‐Phenylalanyl) isolated from *Streptomyces* sp. AMLK‐335. J Microgiol Biotechnol. 2002; 12: 1013–6.

[15] Li J , Wang W , Xu SX , *et al* *Lactobacillus reuteri*‐produced cyclic dipeptides quench agr‐mediated expression of toxic shock syndrome toxin‐1 in staphylococci. Proc Natl Acad Sci USA. 2011; 108: 3360–5. 21282650 10.1073/pnas.1017431108 PMC3044419

[16] Graz M , Hunt A , Jamie H , *et al* Antimicrobial activity of selected cyclic dipeptides. Pharmazie. 1999; 54: 772–5. 10563376

[17] Ström K , Sjögren J , Broberg A , *et al* Lactobacillus plantarum MiLAB 393 produces the antifungal cyclic dipeptides cyclo(L‐Phe‐L‐Pro) and cyclo(L‐Phe‐trans‐4‐OH‐L‐Pro) and 3‐phenyllactic acid. Appl Environ Microbiol. 2002; 68: 4322–7. 12200282 10.1128/AEM.68.9.4322-4327.2002 PMC124062

[18] Ortiz‐Castro R , Díaz‐Pérez C , Martínez‐Trujillo M , *et al* Transkingdom signaling based on bacterial cyclodipeptides with auxin activity in plants. Proc Natl Acad Sci USA. 2011; 108: 7253–8. 21482761 10.1073/pnas.1006740108 PMC3084137

[19] Brauns SC , Milne P , Naudé R , *et al* Selected cyclic dipeptides inhibit cancer cell growth and induce apoptosis in HT‐29 colon cancer cells. Anticancer Res. 2004; 24: 1713–9. 15274345

[20] Brauns SC , Dealtry G , Milne P , *et al* Caspase‐3 activation and induction of PARP cleavage by cyclic dipeptide cyclo(Phe‐Pro) in HT‐29 cells. Anticancer Res. 2005; 25: 4197–202. 16309216

[21] Wieckowski MR , Giorgi C , Lebiedzinska M , *et al* Isolation of mitochondria‐associated membranes and mitochondria from animal tissues and cells. Nat Protoc. 2009; 4: 1582–90. 19816421 10.1038/nprot.2009.151

[22] Kim D , Pertea G , Trapnell C , *et al* TopHat2: accurate alignment of transcriptomes in the presence of insertions, deletions and gene fusions. Genome Biol. 2013; 14: R36. 23618408 10.1186/gb-2013-14-4-r36 PMC4053844

[23] Trapnell C , Hendrickson DG , Sauvageau M , *et al* Differential analysis of gene regulation at transcript resolution with RNA‐seq. Nat Biotechnol. 2013; 31: 46–53. 23222703 10.1038/nbt.2450 PMC3869392

[24] Yen GC , Chiang HC , Wu CH , *et al* The protective effects of Aspergillus candidus metabolites against hydrogen peroxide‐induced oxidative damage to Int 407 cells. Food Chem Toxicol. 2003; 41: 1561–7. 12963009 10.1016/s0278-6915(03)00174-1

[25] Buc E , Dubois D , Sauvanet P , *et al* High prevalence of mucosa‐associated E. coli producing cyclomodulin and genotoxin in colon cancer. PLoS ONE. 2013; 8: e56964. 23457644 10.1371/journal.pone.0056964 PMC3572998

[26] Cheng YT , Wu CH , Ho CY , *et al* Catechin protects against ketoprofen‐induced oxidative damage of the gastric mucosa by up‐regulating Nrf2 *in vitro* and *in vivo* . J Nutr Biochem. 2013; 24: 475–83. 22704780 10.1016/j.jnutbio.2012.01.010

[27] Lee SJ , Jung YH , Oh SY , *et al* *Vibrio vulnificus* VvhA induces NF‐κB‐dependent mitochondrial cell death *via* lipid raft‐mediated ROS production in intestinal epithelial cells. Cell Death Dis. 2015; 6: 1655. 25695598 10.1038/cddis.2015.19 PMC4669806

[28] Lim JG , Choi SH . IscR is a global regulator essential for pathogenesis of *Vibrio vulnificus* and induced by host cells. Infect Immun. 2014; 82: 569–78. 24478072 10.1128/IAI.01141-13 PMC3911388

[29] Dey AK , Bhagat A , Chowdhury R . Host cell contact induces expression of virulence factors and VieA, a cyclic di‐GMP phosphodiesterase, in *Vibrio cholerae* . J Bacteriol. 2013; 195: 2004–10. 23435982 10.1128/JB.02127-12 PMC3624586

[30] Bina XR , Taylor DL , Vikram A , *et al* *Vibrio cholerae* ToxR downregulates virulence factor production in response to cyclo(Phe‐Pro). MBio. 2013; 4: e00366–13. 23982069 10.1128/mBio.00366-13 PMC3760244

[31] Kim K , Kim NJ , Kim SY , *et al* Cyclo(Phe‐Pro) produced by the human pathogen *Vibrio vulnificus* inhibits host innate immune responses through the NF‐κB pathway. Infect Immun. 2015; 83: 1150–61. 25561711 10.1128/IAI.02878-14 PMC4333476

[32] Chiaverini N , De Ley M . Protective effect of metallothionein on oxidative stress‐induced DNA damage. Free Radic Res. 2010; 44: 605–13. 20380594 10.3109/10715761003692511

[33] Vincent‐Hubert F , Châtel A , Gourlay‐Francé C . Metallothionein mRNA induction is correlated with the decrease of DNA strand breaks in cadmium exposed zebra mussels. Mutat Res Genet Toxicol Environ Mutagen. 2014; 766: 10–5. 24681118 10.1016/j.mrgentox.2014.03.006

[34] Kim SM , Kim JY , Lee S , *et al* Adrenomedullin protects against hypoxia/reoxygenation‐induced cell death by suppression of reactive oxygen species *via* thiol redox systems. FEBS Lett. 2010; 584: 213–8. 19932100 10.1016/j.febslet.2009.11.063

[35] Zhu P , Tan MJ , Huang RL , *et al* Angiopoietin‐like 4 protein elevates the prosurvival intracellular O_2_(‐):H_2_O_2_ ratio and confers anoikis resistance to tumors. Cancer Cell. 2011; 19: 401–15. 21397862 10.1016/j.ccr.2011.01.018

[36] Huang CC , Chen KL , Cheung CH , *et al* Autophagy induced by cathepsin S inhibition induces early ROS production, oxidative DNA damage, and cell death *via* xanthine oxidase. Free Radic Biol Med. 2013; 65: 1473–86. 23892358 10.1016/j.freeradbiomed.2013.07.020

[37] Uno S , Sakurai K , Nebert DW , *et al* Protective role of cytochrome P450 1A1 (CYP1A1) against benzo[a]pyrene‐induced toxicity in mouse aorta. Toxicology. 2014; 316: 34–42. 24394547 10.1016/j.tox.2013.12.005

[38] Ros S , Santos CR , Moco S , *et al* Functional metabolic screen identifies 6‐phosphofructo‐2‐kinase/fructose‐2,6‐biphosphatase 4 as an important regulator of prostate cancer cell survival. Cancer Discov. 2012; 2: 328–43. 22576210 10.1158/2159-8290.CD-11-0234

[39] Shen YH , Song GX , Liu YQ , *et al* Silencing of FABP3 promotes apoptosis and induces mitochondrion impairment in embryonic carcinoma cells. J Bioenerg Biomembr. 2012; 44: 317–23. 22528395 10.1007/s10863-012-9439-y

[40] Kim IG , Kim SY , Kim HA , *et al* Disturbance of DKK1 level is partly involved in survival of lung cancer cells *via* regulation of ROMO1 and γ‐radiation sensitivity. Biochem Biophys Res Commun. 2014; 443: 49–55. 24269823 10.1016/j.bbrc.2013.11.038

[41] Shou J , Ali‐Osman F , Multani AS , *et al* Human Dkk‐1, a gene encoding a Wnt antagonist, responds to DNA damage and its overexpression sensitizes brain tumor cells to apoptosis following alkylation damage of DNA. Oncogene. 2002; 21: 878–89. 11840333 10.1038/sj.onc.1205138

[42] Lenaz G . Mitochondria and reactive oxygen species. Which role in physiology and pathology? Adv Exp Med Biol. 2012; 942: 93–136. 22399420 10.1007/978-94-007-2869-1_5

[43] Marchi S , Giorgi C , Suski JM , *et al* Mitochondria‐ros crosstalk in the control of cell death and aging. J Signal Transduct. 2012; 2012: 329635. 22175013 10.1155/2012/329635 PMC3235816

[44] Batandier C , Fontaine E , Kériel C , *et al* Determination of mitochondrial reactive oxygen species: methodological aspects. J Cell Mol Med. 2002; 6: 175–87. 12169203 10.1111/j.1582-4934.2002.tb00185.x PMC6740075

[45] Kudin AP , Bimpong‐Buta NY , Vielhaber S , *et al* Characterization of superoxide‐producing sites in isolated brain mitochondria. J Biol Chem. 2004; 279: 4127–35. 14625276 10.1074/jbc.M310341200

[46] Day BJ , Shawen S , Liochev SI , *et al* A metalloporphyrin superoxide dismutase mimetic protects against paraquat‐induced endothelial cell injury, *in vitro* . J Pharmacol Exp Ther. 1995; 275: 1227–32. 8531085

[47] Gauuan PJ , Trova MP , Gregor‐Boros L , *et al* Superoxide dismutase mimetics: synthesis and structure‐activity relationship study of MnTBAP analogues. Bioorg Med Chem. 2002; 10: 3013–21. 12110324 10.1016/s0968-0896(02)00153-0

[48] Milne PJ , Hunt AL , Rostoll K , *et al* The biological activity of selected cyclic dipeptides. J Pharm Pharmacol. 1998; 50: 1331–7. 10052845 10.1111/j.2042-7158.1998.tb03355.x

[49] Reinhardt HC , Aslanian AS , Lees JA , *et al* p53‐deficient cells rely on ATM‐ and ATR‐mediated checkpoint signaling through the p38MAPK/MK2 pathway for survival after DNA damage. Cancer Cell. 2007; 11: 175–89. 17292828 10.1016/j.ccr.2006.11.024 PMC2742175

[50] Bottero V , Busuttil V , Loubat A , *et al* Activation of nuclear factor kappaB through the IKK complex by the topoisomerase poisons SN38 and doxorubicin: a brake to apoptosis in HeLa human carcinoma cells. Cancer Res. 2001; 61: 7785–91. 11691793

[51] Wei W , Chua MS , Grepper S , *et al* Soluble Frizzled‐7 receptor inhibits Wnt signaling and sensitizes hepatocellular carcinoma cells towards doxorubicin. Mol Cancer. 2011; 10: 16. 21314951 10.1186/1476-4598-10-16 PMC3050858

[52] Anderson RS , Patel KM , Roesijadi G . Oyster metallothionein as an oxyradical scavenger: implications for hemocyte defense responses. Dev Comp Immunol. 1999; 23: 443–9. 10512455 10.1016/s0145-305x(99)00029-4

[53] Reinecke F , Levanets O , Olivier Y , *et al* Metallothionein isoform 2A expression is inducible and protects against ROS‐mediated cell death in rotenone‐treated HeLa cells. Biochem J. 2006; 395: 405–15. 16402917 10.1042/BJ20051253 PMC1422768

[54] Xu J , Wang G , Wang Y , *et al* Diabetes‐ and angiotensin II‐induced cardiac endoplasmic reticulum stress and cell death: metallothionein protection. J Cell Mol Med. 2009; 13: 1499–512. 19583814 10.1111/j.1582-4934.2009.00833.x PMC3828862

[55] Steffens B , Sauter M . Epidermal cell death in rice is confined to cells with a distinct molecular identity and is mediated by ethylene and H_2_O_2_ through an autoamplified signal pathway. Plant Cell. 2009; 21: 184–96. 19141708 10.1105/tpc.108.061887 PMC2648082

[56] Zeitoun‐Ghandour S , Leszczyszyn OI , Blindauer CA , *et al* *C. elegans* metallothioneins: response to and defence against ROS toxicity. Mol BioSyst. 2011; 7: 2397–406. 21647514 10.1039/c1mb05114h

[57] Yoshimoto T , Fukai N , Sato R , *et al* Antioxidant effect of adrenomedullin on angiotensin II‐induced reactive oxygen species generation in vascular smooth muscle cells. Endocrinology. 2004; 145: 3331–7. 15070851 10.1210/en.2003-1583

[58] Liu L , Bridges RJ , Eyer CL . Effect of cytochrome P450 1A induction on oxidative damage in rat brain. Mol Cell Biochem. 2001; 223: 89–94. 11681726 10.1023/a:1017904912654

[59] Jun JI , Lau LF . The matricellular protein CCN1 induces fibroblast senescence and restricts fibrosis in cutaneous wound healing. Nat Cell Biol. 2010; 12: 676–85. 20526329 10.1038/ncb2070 PMC2919364

[60] Bonner WM , Redon CE , Dickey JS , *et al* GammaH2AX and cancer. Nat Rev Cancer. 2008; 8: 957–67. 19005492 10.1038/nrc2523 PMC3094856

[61] Sedelnikova OA , Redon CE , Dickey JS , *et al* Role of oxidatively induced DNA lesions in human pathogenesis. Mutat Res. 2010; 704: 152–9. 20060490 10.1016/j.mrrev.2009.12.005 PMC3074954

[62] Berdelle N , Nikolova T , Quiros S , *et al* Artesunate induces oxidative DNA damage, sustained DNA double‐strand breaks, and the ATM/ATR damage response in cancer cells. Mol Cancer Ther. 2011; 10: 2224–33. 21998290 10.1158/1535-7163.MCT-11-0534

[63] Kowalska‐Loth B , Bubko I , Komorowska B , *et al* Contribution of topoisomerase I to conversion of single‐strand into double‐strand DNA breaks. Mol Biol Rep. 1998; 25: 21–6. 9540064 10.1023/a:1006831527609

[64] Wu J , Yin MB , Hapke G , *et al* Induction of biphasic DNA double strand breaks and activation of multiple repair protein complexes by DNA topoisomerase I drug 7‐ethyl‐10‐hydroxy‐camptothecin. Mol Pharmacol. 2002; 61: 742–8. 11901212 10.1124/mol.61.4.742

[65] Pommier Y , Redon C , Rao VA , *et al* Repair of and checkpoint response to topoisomerase I‐mediated DNA damage. Mutat Res. 2003; 532: 173–203. 14643436 10.1016/j.mrfmmm.2003.08.016

[66] Kim IH , Son JS , Wen Y , *et al* Transcriptomic analysis of genes modulated by cyclo(L‐phenylalanine‐L‐proline) in *Vibrio vulnificus* . J Microbiol Biotechnol. 2013; 23: 1791–801. 24100622 10.4014/jmb.1308.08068

[67] Cuevas‐Ramos G , Petit CR , Marcq I , *et al* Escherichia coli induces DNA damage *in vivo* and triggers genomic instability in mammalian cells. Proc Natl Acad Sci USA. 2010; 107: 11537–42. 20534522 10.1073/pnas.1001261107 PMC2895108

[68] Sun G , Xu X , Wang Y , *et al* *Mycoplasma pneumoniae* infection induces reactive oxygen species and DNA damage in A549 human lung carcinoma cells. Infect Immun. 2008; 76: 4405–13. 18663006 10.1128/IAI.00575-08 PMC2546820

[69] Chumduri C , Gurumurthy RK , Zadora PK , *et al* Chlamydia infection promotes host DNA damage and proliferation but impairs the DNA damage response. Cell Host Microbe. 2013; 13: 746–58. 23768498 10.1016/j.chom.2013.05.010

[70] Toller IM , Neelsen KJ , Steger M , *et al* Carcinogenic bacterial pathogen *Helicobacter pylori* triggers DNA double‐strand breaks and a DNA damage response in its host cells. Proc Natl Acad Sci USA. 2011; 108: 14944–9. 21896770 10.1073/pnas.1100959108 PMC3169107

