# Supplementary figures and images for: Cyclo(phenylalanine‐proline) induces DNA damage in mammalian cells via reactive oxygen species
Source: J Cell Mol Med. 2015 Sep 28;19(12):2851–64. doi: 10.1111/jcmm.12678 (PMC4687708; doi:10.1111/jcmm.12678)

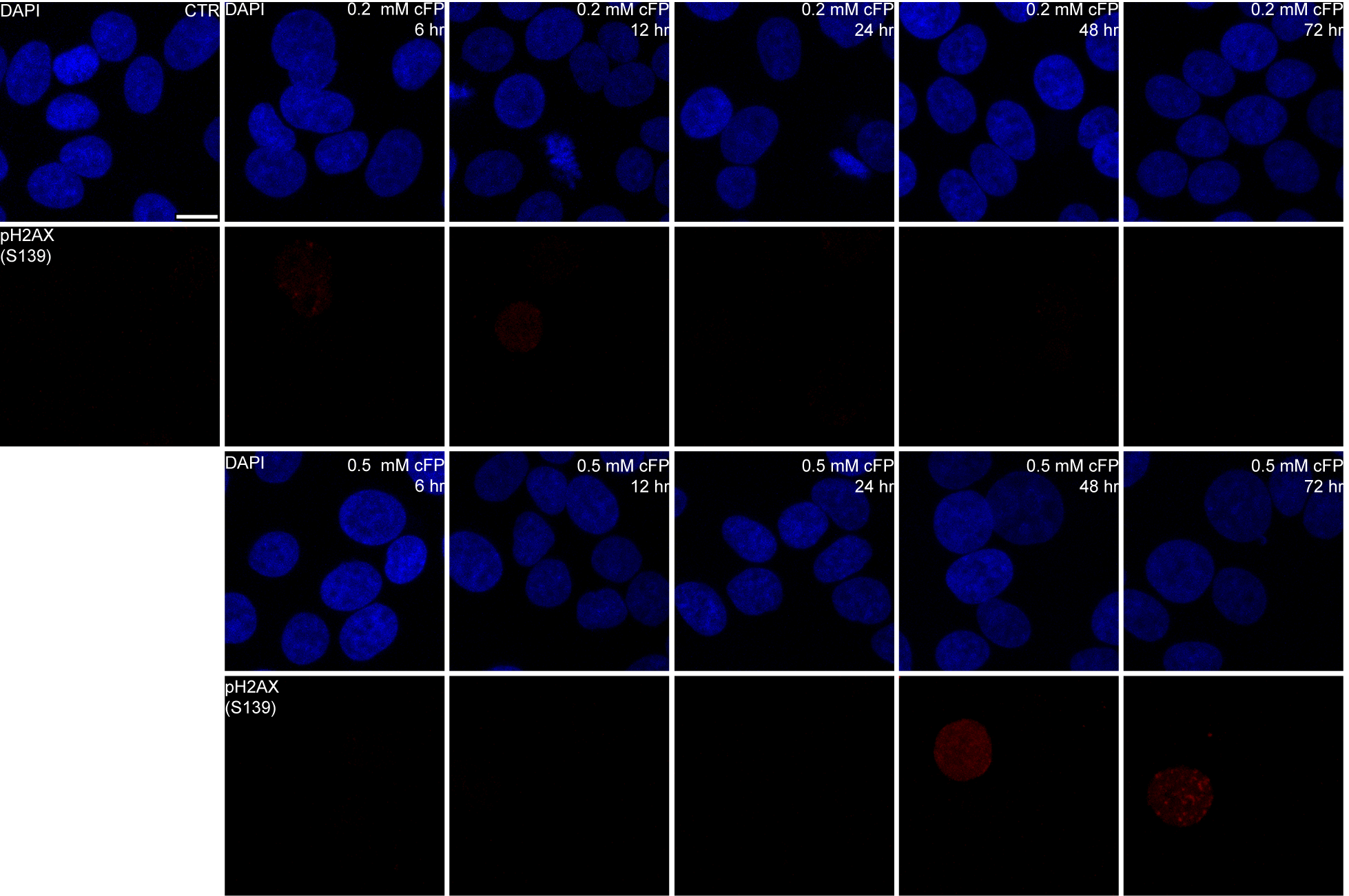

Supplement: Supplementary file 1 — Figure S1 Induction of DNA damage in cFP‐treated INT 407 cells. [file JCMM-19-2851-s001.tif]

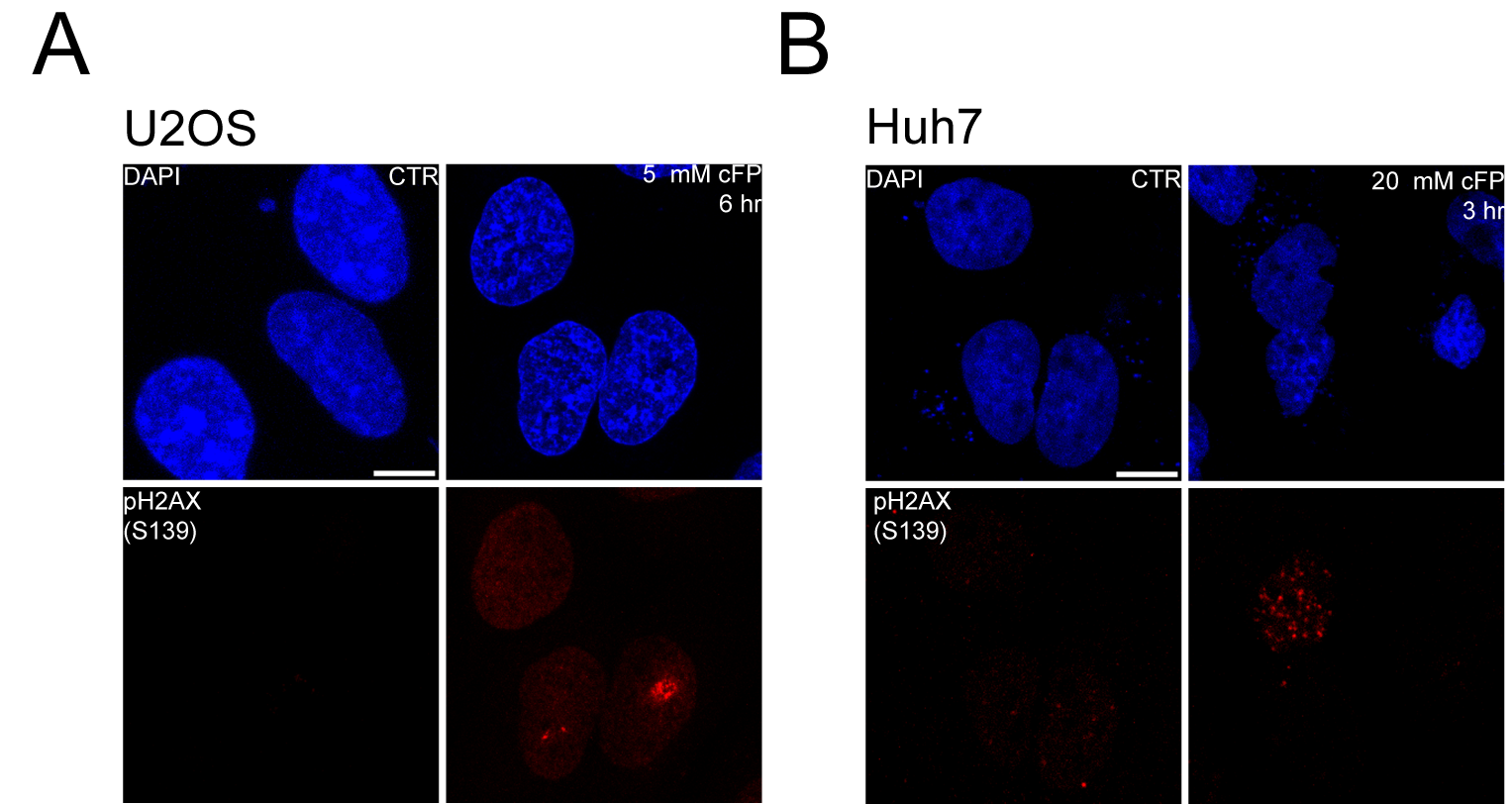

Supplement: Supplementary file 2 — Figure S2 Induction of DNA damage in 5 or 20 mM cFP‐treated U2OS osteosarcoma cells and Huh7 hepatoma cells respectively. [file JCMM-19-2851-s002.tif]

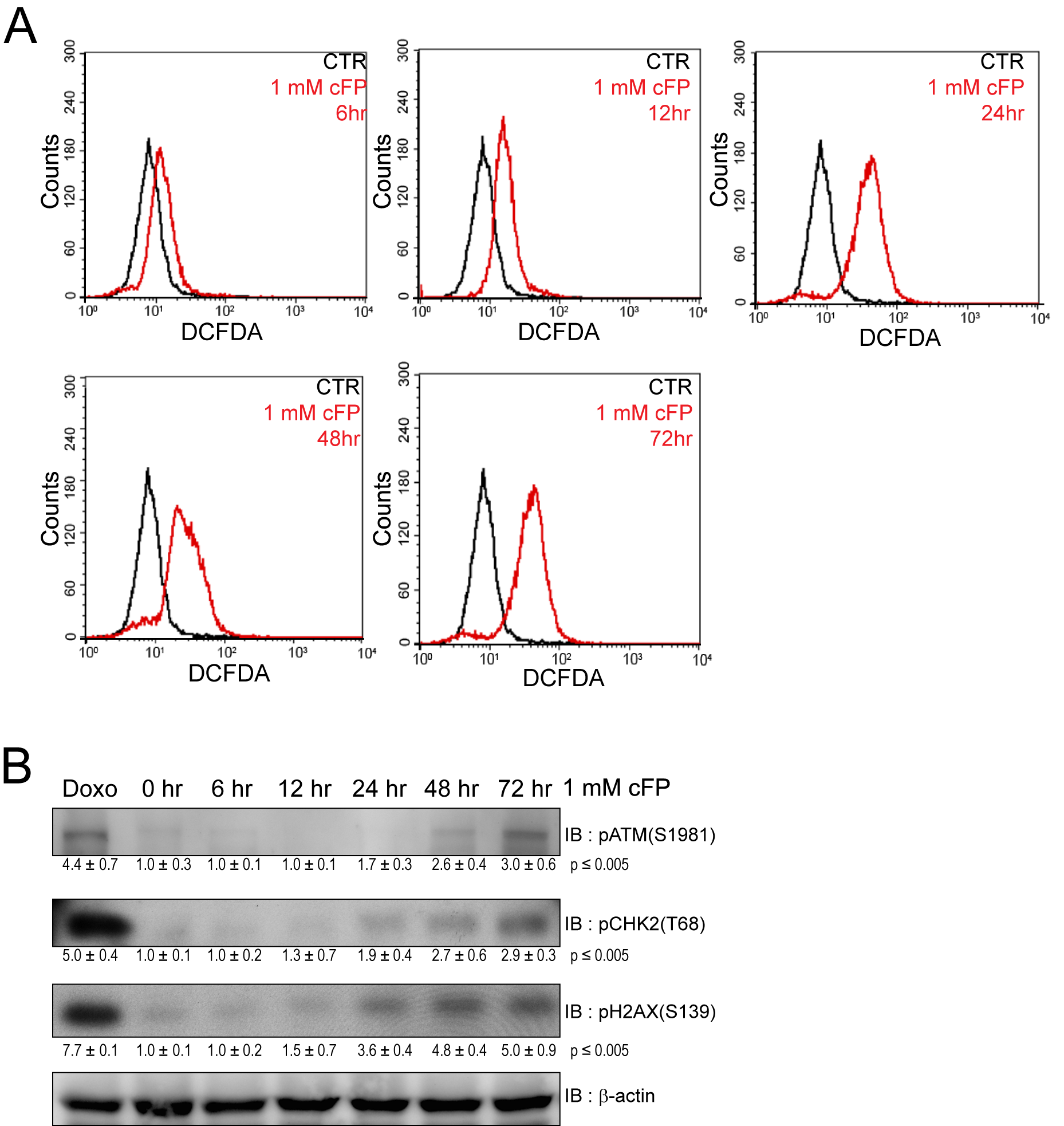

Supplement: Supplementary file 3 — Figure S3 Incubation time‐dependent ROS production and DNA damage in 1 mM cFP‐treated INT‐407 cells. [file JCMM-19-2851-s003.tif]

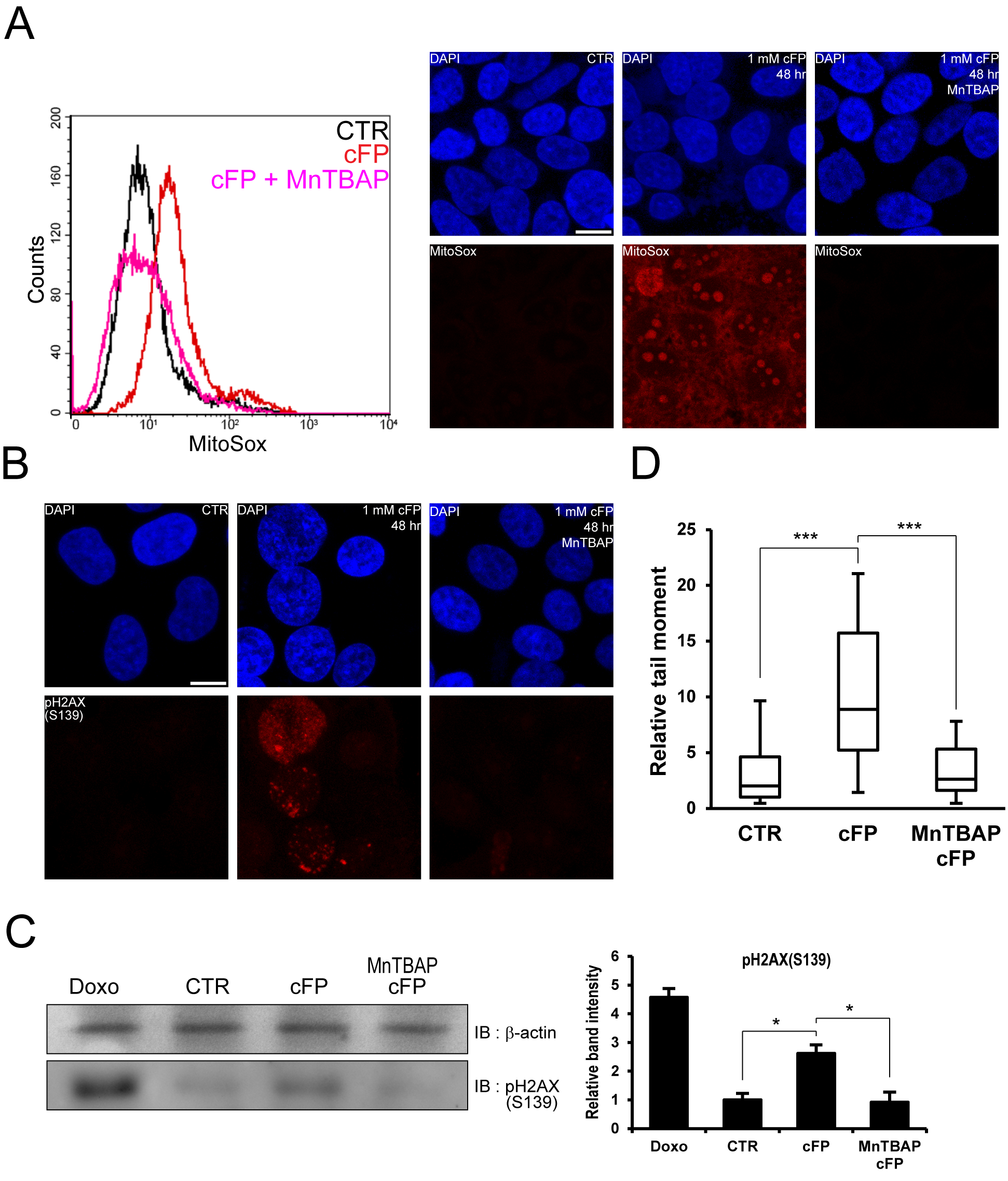

Supplement: Supplementary file 4 — Figure S4 Superoxide‐dependent DNA damage in 1 mM cFP‐treated INT‐407 cells. [file JCMM-19-2851-s004.tif]

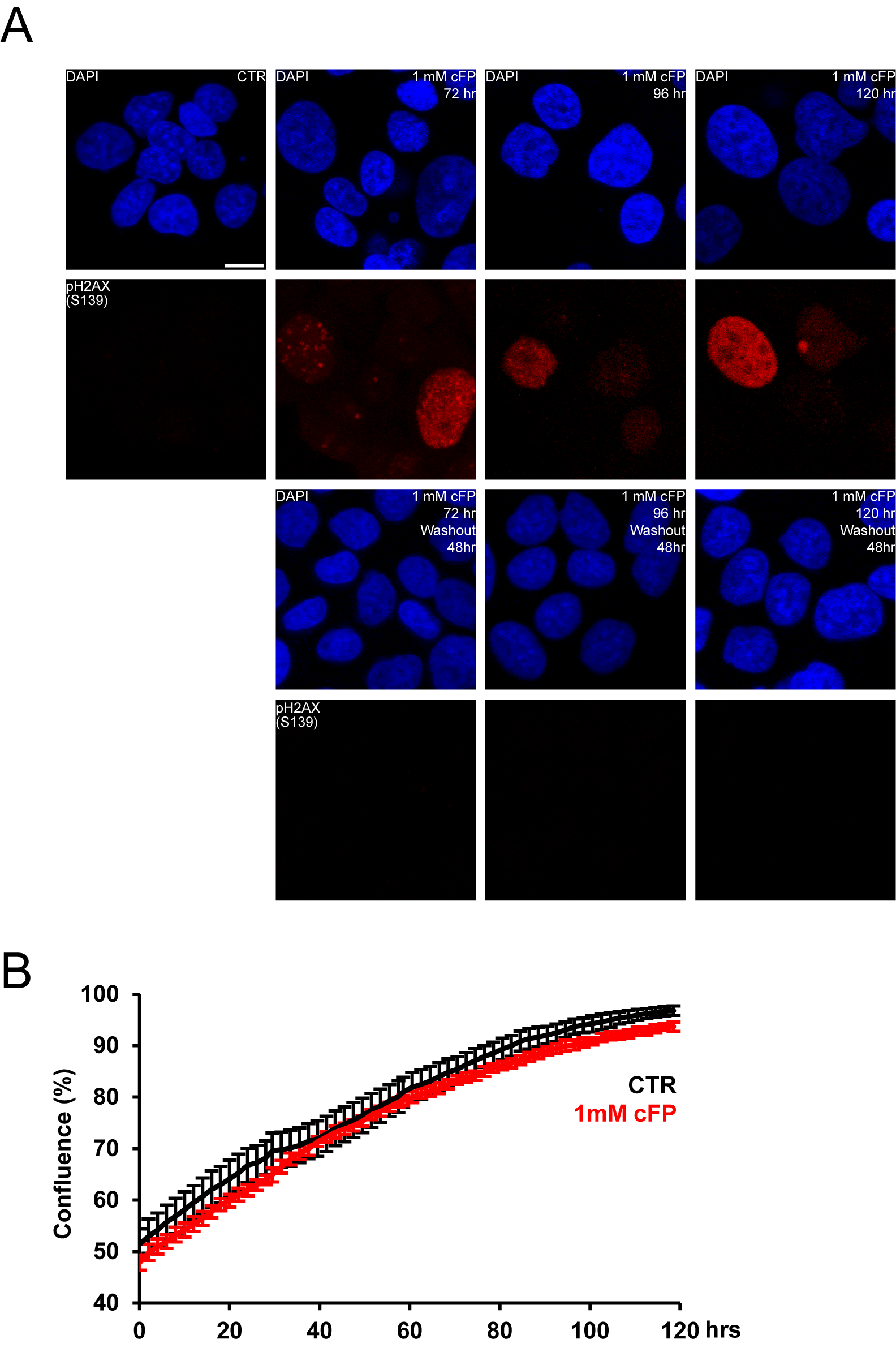

Supplement: Supplementary file 5 — Figure S5 Incubation time‐independent restoration of DNA damage repair after removal of cFP. [file JCMM-19-2851-s005.tif]

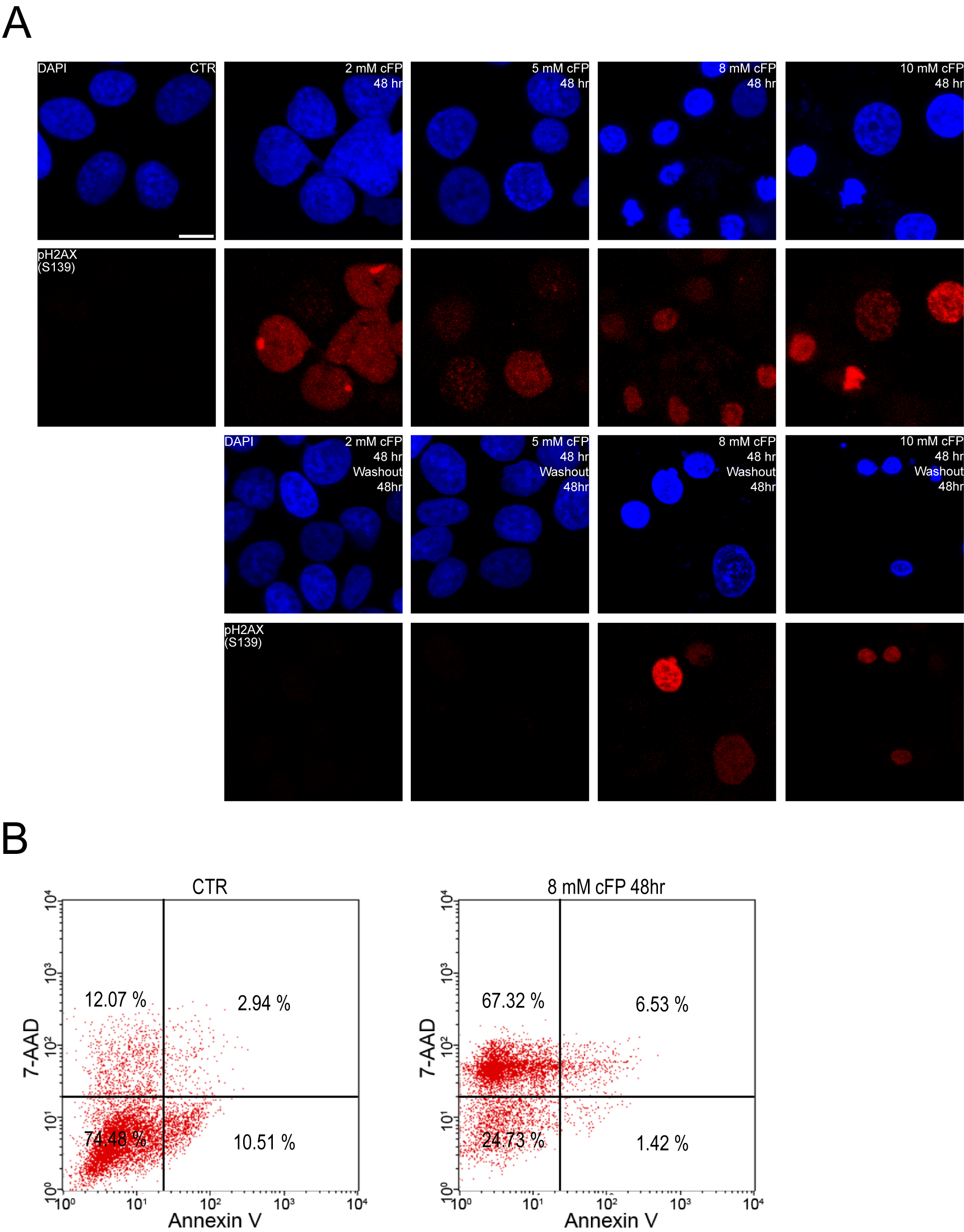

Supplement: Supplementary file 6 — Figure S6 Dose‐dependent restoration of DNA damage repair after removal of cFP. [file JCMM-19-2851-s006.tif]
