# Supplementary material for: Cyclo(phenylalanine‐proline) induces DNA damage in mammalian cells via reactive oxygen species
Source: J Cell Mol Med. 2015 Sep 28;19(12):2851–64. doi: 10.1111/jcmm.12678 (PMC4687708; doi:10.1111/jcmm.12678)
Supplement: Supplementary file 7 — Table S1 Oligonucleotides used in this study for real‐time PCR. [file JCMM-19-2851-s007.docx]

Table S1. Oligonucleotides used in this study for real-time PCR

| **Gene name** | **Forward Primer** | **Reverse Primer** | **Length**  **(bp)** |
| --- | --- | --- | --- |
| ***CYR61*** | **CGCCTTGTGAAAGAAACCCG** | **GGTTCGGGGGATTTCTTGGT** | **100** |
| ***CYP1A1*** | **CAAGAGGAGCTAGACACAGTGATT** | **AGCCTTTCAAACTTGTGTCTCTTGT** | **166** |
| ***ALDOC*** | **GCCAAATTGGGGTGGAAAACA** | **TTCACACGGTCATCAGCACTG** | **79** |
| ***CA9*** | **CTGTCTCGCTTGGAAGAAATCG** | **GCAGGAGTGCAGATATGTCCAG** | **76** |
| ***ADM*** | **TGCCCAGACCCTTATTCGG** | **AGTTGTTCATGCTCTGGCGG** | **116** |
| ***GBP1*** | **CCAGTTGCTGAAAGAGCAAGAGA** | **TCCCTCTTTTAGTAGTTGCTCCTGTT** | **73** |
| ***MT2A*** | **GCAAATGCACCTCCTGCAAG** | **GTGGAAGTCGCGTTCTTTAC** | **161** |
| ***FABP3*** | **CACTCACCCACGGCACTGCA** | **TCCCGGTCAGTGGCACCTGA** | **187** |
| ***DKK1*** | **GGGAATTACTGCAAAAATGGAATA** | **ATGACCGGAGACAAACAGAAC** | **190** |
| ***MT1X*** | **TCATCTGTCCCGCTGCGTGT** | **GCGAGCAGGAGCAGTTGGGG** | **86** |
| ***CTSS*** | **TGACAACGGCTTTCCAGTACA** | **GGCAGCACGATATTTTGAGTCAT** | **113** |
| ***ANGPTL4*** | **GCTGCATGCGTTGCCTC** | **CCCTTGGTCCACGCCTCTA** | **69** |
| ***PFKFB4*** | **CAACATCGTGCAAGTGAAACTG** | **GACTCGTAGGAGTTCTCATAGCA** | **11** |
| ***ATM*** | **ATCTGCTGCCGTCAACTAGAA** | **GATCTCGAATCAGGCGCTTAAA** | **82** |
| ***GAPDH*** | **AGGTCGGTGTGAACGGATTTG** | **TGTAGACCATGTAGTTGAGGTCA** | **123** |
